# Improving the identification and management of chronic kidney disease in primary care: lessons from a staged improvement collaborative

**DOI:** 10.1093/intqhc/mzu097

**Published:** 2014-12-18

**Authors:** Gill Harvey, Kathryn Oliver, John Humphreys, Katy Rothwell, Janet Hegarty

**Affiliations:** 1 Manchester Business School, University of Manchester, Booth Street West, Manchester M15 6PB, UK; 2 School of Nursing, University of Adelaide, Eleanor Harrald Building, Frome Road, Adelaide SA5005, Australia; 3 School of Social Sciences, University of Manchester, Arthur Lewis Building, Oxford Road, Manchester M13 9PL, UK; 4 Department for Science, Technology, Engineering and Public Policy (UCL STEaPP), University College London, 36-38 Fitzroy Square (2nd Floor), London W1T 6EY, UK; 5 NIHR CLAHRC for Greater Manchester, 3rd Floor, Mayo Building, Salford Royal NHS Foundation Trust, Stott Lane, Salford M6 8HD, UK; 6 Renal Department, Salford Royal NHS Foundation Trust, Stott Lane, Salford M6 8HD, UK

**Keywords:** improvement collaborative, chronic kidney disease, primary care, implementation, evidence-based guidance

## Abstract

**Quality problem:**

Undiagnosed chronic kidney disease (CKD) contributes to a high cost and care burden in secondary care. Uptake of evidence-based guidelines in primary care is inconsistent, resulting in variation in the detection and management of CKD.

**Initial assessment:**

Routinely collected general practice data in one UK region suggested a CKD prevalence of 4.1%, compared with an estimated national prevalence of 8.5%. Of patients on CKD registers, ∼30% were estimated to have suboptimal management according to Public Health Observatory analyses.

**Choice of solution:**

An evidence-based framework for implementation was developed. This informed the design of an improvement collaborative to work with a sample of 30 general practices.

**Implementation:**

A two-phase collaborative was implemented between September 2009 and March 2012. Key elements of the intervention included learning events, improvement targets, Plan-Do-Study-Act cycles, benchmarking of audit data, facilitator support and staff time reimbursement.

**Evaluation:**

Outcomes were evaluated against two indicators: number of patients with CKD on practice registers; percentage of patients achieving evidence-based blood pressure (BP) targets, as a marker for CKD care. In Phase 1, recorded prevalence of CKD in collaborative practices increased ∼2-fold more than that in comparator local practices; in Phase 2, this increased to 4-fold, indicating improved case identification. Management of BP according to guideline recommendations also improved.

**Lessons learned:**

An improvement collaborative with tailored facilitation support appears to promote the uptake of evidence-based guidance on the identification and management of CKD in primary care. A controlled evaluation study is needed to rigorously evaluate the impact of this promising improvement intervention.

## Outline of problem

Chronic kidney disease (CKD) is a progressive loss of kidney function over a period of at least 3 months. Since 2002, a system of classifying CKD according to five stages has been applied [1] (see Table 1). Many patients with CKD Stages 3–5 are not identified until the disease is advanced. 1.3 and 19.9% of patients at Stages 3 and 4, respectively, will progress to end-stage kidney disease (ESKD) within 5 years [2]. This is costly for health care systems and patients alike, with ESKD requiring a burdensome and costly treatment programme in secondary care. Asymptomatic patients at all (including the early) stages of disease also have an increased risk of hospitalization through both cardiovascular events and acute kidney injury [3]. The prevalence of CKD in the adult population is debated; estimates in the UK suggest a population average between 6.76 and 8.5% [4, 5], a figure that varies according to age, sex, ethnicity and co-morbidity. Prevalence increases exponentially with age; thus with current demographic trends, CKD is likely to become an increasingly important public health issue.

**Table 1:** Classification of stages for CKD [1]

Stage of CKD	Description	Glomerular filtration rate (GFR)
1.	Kidney damage with normal or raised GRF	>90
2.	Kidney damage with mildly reduced GFR	60–89
3.	Moderately reduced GRF	30–59
4.	Severe reduction in GFR	15–29
5.	Kidney failure	<15

National and international evidence-based clinical guidelines have been developed to promote the identification and improved management of CKD Stages 3–5 [1, 6]. In the UK, case finding for CKD has also been financially incentivized since 2006 through a primary care pay-for-performance scheme known as the Quality and Outcomes Framework (QOF) [7]. These initiatives recognize the potential for improving the diagnosis and management of CKD patients in primary care, in terms of enhancing patient outcomes and quality of life and realizing significant cost savings for health care systems in the longer term. Despite these initiatives, identification of early-stage CKD in primary care remains inconsistent [8].

## Initial assessment

The project was undertaken in Greater Manchester, a region in the north of England with a poor overall life expectancy [9]. In 2008, the National Institute for Health Research (NIHR) Collaboration for Leadership in Applied Health Research and Care (CLAHRC) Greater Manchester was established with the aim of reducing inequalities in vascular disease through conducting and implementing applied health research. CKD was identified as one of the areas to be addressed within this programme of work. QOF data for 2008/09 recorded the prevalence of CKD in Greater Manchester as 4.1% [10], considerably lower than national population estimates, based on available research evidence, of ∼8.5% [4]. Of the patients identified on general practice registers, QOF data showed that ∼30% had suboptimal management, defined as uncontrolled or unknown blood pressure (BP) [10].

These data demonstrated the potential for improvement as although patients with CKD are often asymptomatic, they can be identified reasonably easily through routine clinical tests, such as blood biochemistry and urinalysis. Moreover, they often have co-morbidities and are on other primary care disease registers. The challenge was to find a way to effectively translate the available evidence on identifying and managing CKD into primary care practice (see Box 1).

Box 1 **Guideline recommendations for the identification and management of adult patients with CKD in primary care**

In primary care in the UK, NICE CKD guidelines (2008) recommend that:

–People should be offered testing for CKD whether they have risk factors such as diabetes, hypertension, cardiovascular disease, family history of Stage 5 CKD or hereditary kidney disease or whether there is opportunistic detection of haematuria or proteinuria;

–Testing should include obtaining a minimum of three estimated glomerular filtration rates (eGFR) over a period of <90 days;

–Identified CKD patients coded at Stage 3A/B should be monitored every 6 months in general practice by eGFR testing. This is adjusted to every 3 months or every 6 weeks, if the patient is coded at Stages 4 or 5, respectively;

–For CKD patients without proteinuria, BP should be managed within the range <140/90; for patients with proteinuria, the range should be <130/80;

–Stable Stage 4 cases should ideally be managed within primary care, whereas more complicated cases and those at Stage 5 should be monitored by renal specialists;

–Proteinuria status and BP should be monitored for all CKD patients at least annually.

Source: NICE (2008) [6].

## Choice of solution

An evidence-based implementation framework was developed, drawing upon available research about effective strategies for implementing evidence-based recommendations into practice. This framework comprised a number of elements, which have previously been described [11]. Briefly, they comprised a conceptual framework – the Promoting Action on Research Implementation in Health Services framework [12] – which proposes that successful implementation is dependent on the nature of the evidence to be implemented, the context in which implementation takes place and the way in which the process is facilitated. Within these conceptual co-ordinates of evidence, context and facilitation, a modified version of the Model for Improvement was identified as an operational framework to guide implementation, with its emphasis on establishing and measuring targets for improvement and using Plan-Do-Study-Act (PDSA) cycles to work towards established goals [13]. In order to support the implementation process, two additional elements were incorporated: multi-disciplinary teams made up of improvement facilitators, project managers, information specialists, clinicians and academics to plan, lead and support the improvement programme; an embedded approach to evaluation and learning to ensure ongoing reflection on and refinement of the improvement programme.

Following stakeholder consultation within the primary care setting, an improvement collaborative (see Box 2) was selected as an appropriate way to structure the planned project. This was seen to be relevant for a number of reasons: first, the need to work with multiple general practices at the same time; second, the fact that collaborative methodology builds on the Model for Improvement [15]; third, a recognition of the potential benefits to be achieved through bringing members of different general practice teams together to learn and share experiences.

Box 2 **Description of the improvement collaborative approach [14]**

- Based on the Institute for Healthcare Improvement breakthrough series approach;
- Brings together subject matter experts and practitioners to test and implement changes in care within a structured, experiential learning framework;
- Teams commit to working on a common topic over a period of ∼12 months, with shared goals and an agreed measurement strategy;
- Teams alternate between shared learning events (to exchange ideas and encourage learning across teams) and action periods (using the Model for Improvement to test small ideas in the workplace);
- An agreed change package is used, with regular monitoring and feedback of progress against targets.

## Implementation

The first step involved establishing an expert faculty to review the evidence on CKD and agree the scope and aims of the collaborative. The faculty included clinical staff from primary and secondary care, patient representatives and individuals with expertise in improvement. Two overarching objectives were established: to halve the gap between recorded and estimated prevalence and to ensure that 75% patients with recorded CKD had their BP managed according to the targets recommended in national guidelines [6] (<140/90 for patients without proteinuria; <130/80 for patients with proteinuria). BP management was identified as the measure reflecting the overall quality of CKD care as it links to progression of CKD and cardiovascular outcomes.

### Phase 1 collaborative

Nineteen practices from four administrative areas within Greater Manchester took part in Phase 1. The project budget was sufficient to support involvement of 4–5 practices per area (representing 7–10% of the practice population). Practices were recruited by a mix of self-selection following advertisement of the initiative and nomination by local NHS managers. Recruited practices represented different sizes (as measured by patient 18+ list size, range 1671–9974) and adult CKD prevalence at baseline (range 1.5–5.9%). Once enrolled, practices were invited to the first of three joint learning events. Each practice was encouraged to send three staff members who would form the practice improvement team. To reflect the multi-disciplinary nature of the project, it was suggested that the improvement team should include General Practitioner, nursing and administrative representation. The first learning event introduced the improvement collaborative methodology, CKD guidelines and improvement targets for the collaborative. Subsequent learning events addressed issues such as building effective improvement teams, creating a receptive context for change and sustaining improvements in practice. Between the learning events, improvement teams were expected to test and apply planned changes using PDSA cycles. Two facilitators made regular practice visits to support the improvement process and provide help with activities such as data searches, managing practice registers, developing process maps and advising on how to overcome particular barriers or problems. This latter activity was informed by an initial assessment of the practice context to identify factors that could facilitate or impede improvement, for example, the way in which the practice was usually run, issues of leadership and culture, the level of teamwork and communication.

All practices were required to submit monthly data relating to the numbers of patients on the CKD register, numbers tested for proteinuria and the number of patients with BP managed to target. These data were analysed by the project team and reported back to practices on a monthly basis in the form of run charts, which presented progress against the key indicators over time both for their own practice and the collaborative as a whole. This allowed individual practices to benchmark progress against their peers; it also enabled the improvement facilitators to identify areas where more targeted input and support was required.

At project inception stakeholder input had indicated that uptake of the improvement programme would only be possible with staff time reimbursement. As part of the implementation strategy, therefore, practices received financial resources to secure protected time for improvement teams; different practices spent this in different ways including locum doctor cover to release physicians for improvement work and buying additional hours from practice staff. In addition in Phase 1, a small additional per patient payment was made for completing key stages of the improvement process (baseline data collection and attendance at the three learning sessions).

### Moving from Phase 1 to Phase 2

At the end of Phase 1, a closing meeting was held to share the experiences and successes of the participating practices. The facilitation team also reflected on the learning gained during the collaborative, informed by the formative evaluation that had been ongoing throughout the implementation process (and which is detailed more fully in the following section). This led to the development of a CKD improvement guide, which summarized the key activities involved in implementation: creating a foundation for improvement, identifying patients with CKD, achieving optimal management of patients with CKD and ensuring improvements are sustained [14]. This guide, complete with accompanying resources and links to other useful available information, was made available as an online resource [16]. It also led to discussions with a CLAHRC initiative in another region of the country that had been involved in developing a data extraction and audit tool to analyse practice registers in relation to patients with CKD. The two CLAHRCs agreed to collaborate to develop a programme known as IMPAKT™ (IMproving Patient care and Awareness of Kidney disease progression Together).

### Phase 2 collaborative

The IMPAKT^™^ tool [17] formed a central part of the evidence and implementation resource for Phase 2 of the improvement collaborative. Other changes from Phase 1 included the appointment of a practice nurse from one of the practices that had been involved in the Phase 1 collaborative to work as a facilitator within the CLAHRC team. This brought the benefits of someone with insider knowledge of the practice environment and the improvement process, as well as local political knowledge and longstanding local professional relationships. Due to financial constraints within the health care sector and the wider CLAHRC programme, the payments available to participating practices were reduced and the collaborative learning events were reduced from three events to two and from a full to half day. Additional opportunities for meeting and sharing experiences were, however, provided through five WebEx seminars scheduled at the outset of the project.

Selection was different in Phase 2 as 10 of the 11 practices were from the same geographical and administrative area of Greater Manchester (which had 5 practices involved in the first collaborative and from where the practice nurse facilitator was seconded). This was a deliberate strategy building on the local profile of the initiative and utilizing the seconded facilitator's pre-existing relationships to assist with engagement. The facilitators liaised with in-house information technology experts to install IMPAKT™ and then worked with practices to interpret and use data from the tool, as part of the process of interrogating and verifying practice registers to identify patients with CKD. As in Phase 1, once patients with CKD were identified, the focus turned to achieving optimal patient management.

## Evaluation

**Figure 1:**
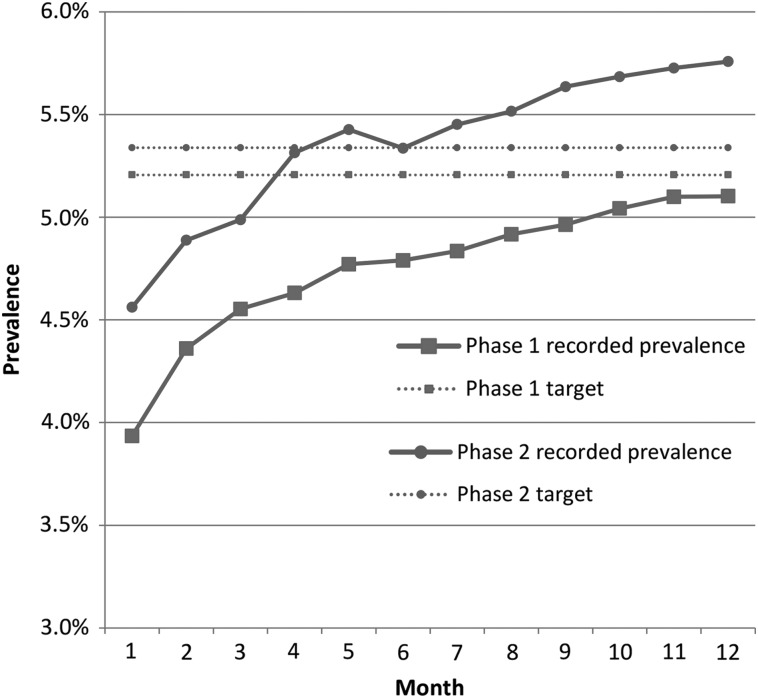
Change in recorded prevalence by month.

**Figure 2:**
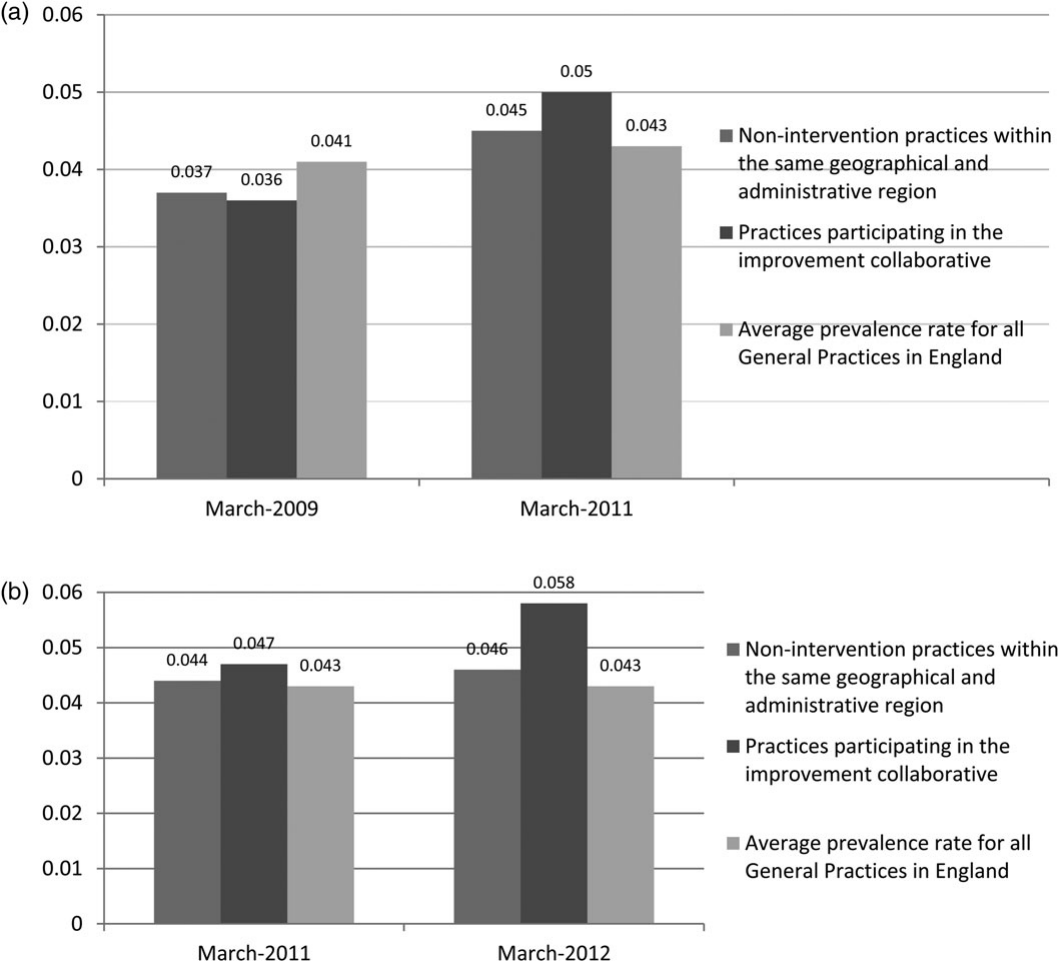
Comparison of change in prevalence rates over time: intervention vs. non-intervention sites. (**a**) Phase 1 collaborative (QOF reporting is within annual period from April to March. The Phase 1 collaborative ran from September 2009 to 2010; therefore, the comparison with non-collaborative practices is made over the two relevant QOF cycles of reporting). (**b**) Phase 2 collaborative.

Each practice had its own prevalence target to achieve, depending on its starting position and an estimated prevalence value, which was modelled on a practice by practice basis using research evidence produced by Public Health England [18]. Overall, the number of patients on CKD registers increased by 1863. In both Phases 1 and 2, there was a recorded 1.2% increase in prevalence. In Phase 1, 1324 additional patients were added to registers; this represented 92% achievement of the original aim set (*n* = 1441) to halve the gap between recorded and estimated prevalence of CKD. Ten of the 19 practices achieved the target. In Phase 2, 539 additional patients were identified, equivalent to 154% achievement of the target. Overall, the target was achieved by Month 3, and 7 of the 11 participating practices reached the target by the close of the project (see Fig. 1). Comparing Phase 1 with 2, the baseline prevalence in Phase 1 was lower than that in Phase 2, which in turn made the average prevalence increase to be achieved greater (2.5% compared with 1.4%). In order to assess the improvements made in the collaborative sites, we compared their pre- and post-intervention prevalence rates with QOF data over the same time periods for non-collaborative sites within the same area and with national averages (see Figs 2a and b). These data indicate improved identification of patients with CKD in collaborative practices compared with both local and national comparators. Interestingly, in the first time period, there were general increases in prevalence, most likely driven by the pay-for-performance system and stepwise improvements in awareness of CKD. The collaborative practices still demonstrated an approximate 2-fold increase in CKD prevalence compared with local comparators. During 2011–12, there was little or no change in prevalence achieved locally or nationally (Fig. 2b), but the improvement practices showed a 4-fold increase in their CKD prevalence.

**Figure 3:**
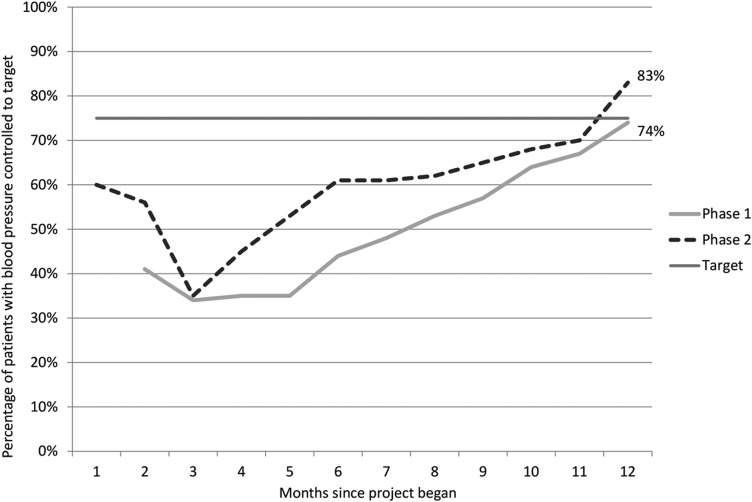
Percentage of CKD patients with blood pressure managed to NICE targets by month.

In relation to the second aim, management of BP improved in both phases; from 34 to 74% of patients managed to NICE targets in Phase 1 and an increase from 60 to 83% in Phase 2 (see Fig. 3). Nine of the 19 practices achieved the 75% target in Phase 1 and all 11 practices reached it in Phase 2. In order to assess the progress made by collaborative practices, we analysed the QOF pay-for-performance data from the geographical region that had the greatest level of involvement in Phases 1 and 2 of the project. The actual timeframes for QOF reporting do not exactly match the time periods during which the collaboratives took place; however, looking over the total timeframe of the project, there is evidence to suggest that collaborative practices reported better levels of BP management in their CKD population (see Table 2).

**Table 2:** Comparison of diagnosed CKD patients within one geographical region treated to national pay-for-performance (QOF) blood pressure targets by involvement in the improvement collaborative

Time period	% achievement of target BP in CKD collaborative practices	% achievement of target BP in non-CKD collaborative practices
2010–11	83	74
2011–12	82	73
2012–13	82	75

The difference between the QOF and NICE blood pressure targets was that testing for proteinuria was a pre-requisite of meeting the NICE target. This accounts for the lower baseline figures reported in the collaborative sites when comparing against NICE targets.

Despite the overall level of achievement being close to or above the targets set, variation between practices occurred as apparent from the number of practices that failed to achieve specific targets, particularly in Phase 1. The process evaluation helped to illuminate the factors that contributed to the observed variation. In Phase 1, we analysed the process of implementation through a number of different approaches: monthly review of individual practices at project team meetings, informal interviews and discussions with practice staff during practice-facilitator meetings and asking practices to review and rate their own progress at the learning events. In Phase 2, the ongoing formative evaluation was supplemented by a more in-depth qualitative study in the final stages of the collaborative (January to April 2012) to gain greater insight into the mechanisms and processes that contributed to project outcomes [19].

Common enabling factors included the learning events, the support of the facilitators, having clearly defined targets, regular feedback of data and financial support to participate in the collaborative. A specific barrier that many practices faced in Phase 1 was difficulty extracting the required data on CKD from practice registers, due to a lack of knowledge and skills or problems with the system itself. This was something that the introduction of IMPAKT™ helped to overcome. Other factors could act as an enabler (if present) or a barrier (if absent) and appear to account for the variation that was observed in achievement of the collaborative goals. These included the priority attached to CKD as a topic for improvement; the support of senior leadership; the receptivity of the practice context to new ideas and change; the extent of wider staff engagement in the improvement project, beyond the designated improvement team (see Box 3 for illustrative quotes).

Box 3 **Qualitative experiences of participants in the Phase 2 collaborative**

Enablers of improvement:

‘We had the long first session, which was great, but for the other people (the facilitator) did a half an hour with them to get them up to speed with what was going on… . We could have probably done this ourselves, but having someone come in from outside made it clearer to them that it wasn't just something that we decided to do on a whim, but that it was actually part of a bigger project we were involved in’. (Clinical coordinator)

‘(The support has been) excellent. I've really enjoyed it. I have found the support excellent from both of them. I am the only practice nurse in the practice, so it's been nice that (the clinical facilitator) has come in from a practice nurse point of view and she's been a fantastic support. Really, in a way, it's a bit like clinical supervision as well because we've discussed other things’. (Practice Nurse)

‘I feel that with the support I've had, I am more confident talking to patients. When we sent the initial letters out some patients were coming in and saying I haven't got kidney disease. It hadn't been discussed with them previously. So that needed a little bit of work around and explaining about what chronic means because a lot of people thought it meant severe, whereas to us it's chronic, long term’. (Practice Nurse)

‘There's a kind of structure to it and I think it needs that really. You need to have certainty to get the building blocks in place. But, I think that once you've got those building blocks in place and because of the knowledge that you get over time going through the year, when you come to sort of taking things on yourself, it's all in place for you to continue’. (GP)

Barriers to improvement:

‘It was the initial education and time needed to get the register validated and then put a protocol together (that took the time)… . But that initial work is done now, it wouldn't need to be repeated again and it's just a routine thing that everyone does now, so it's paid off in the end’. (Practice Nurse)

‘I tried to coordinate (the project) really, blocking out time so that people had time to devote to it. There was a bit of a battle over the time at first and management just thought that it could just happen in clinic time and we have had to fight for that’. (Practice Administrator)

‘If I was ever involved in a project like this again, it's one of the things that I would really stick my neck out on is that the rest of the practice wasn't involved early enough. If we'd had more involvement from the others earlier it would have saved us a lot of work because a lot of the results and things that were coming in that we'd instigated weren't necessarily coming through to us so again, they were being filed as normal or weren't being coded. It was then just double work’. (Practice Nurse)

‘It's no use (the practice nurse) and I going through all these codes if the rest of the practice (team) aren't going to do it. For me that was the most important thing. It can't just be two people doing it. It has to be the whole practice staff… . Every single person that sees patients needs to have some grasp of what's going on, even to the point of the admin staff because they are sending out letters for annual reviews’. (GP)

## Lessons learned

Through working together on the improvement of CKD over a period of 30 months, a number of important lessons emerged. First, the application of an improvement collaborative methodology, informed by an evidence-based implementation framework, appears to have enabled increased identification and improved management of patients with CKD in primary care, over and above that which could be attributed to the national pay-for-performance system (QOF). Key features of the improvement collaborative (shared learning and networking, clear improvement targets, regular audit and feedback) plus a dedicated practice facilitator providing tailored support appear to act as important enablers of improvement. This concurs with findings from other studies of practice facilitation; a recent systematic review suggests that practices supported by a facilitator are almost three times more likely to implement clinical guidelines [20].

Second, our experience highlights the benefits of adopting a longitudinal approach to improvement work in terms of achieving greater efficiencies and accelerating the rate of improvement. Through the course of running two sequential collaboratives, we have accumulated learning about the management of CKD, resulting in the development and continuous refinement of practical resources to be used by improvement teams and a greater understanding of the contextual variables that need to be addressed to facilitate more tailored – and ultimately more successful – implementation.

Third, we have begun to develop an external–internal model of facilitation within local primary care practice. We believe this is an important area for future development to build capacity for improvement within primary care and enhance sustainability over the longer term. Practice-based facilitation is an area of growing interest and activity [21, 22] and one that clearly involves a substantive investment in terms of people, time and resources. There are many questions still to be answered to better understand the appropriate balance between investment costs and benefits realization, for example: who should take on the facilitator role? How best to identify, prepare, develop and support facilitators? What dose and frequency of facilitator intervention works best? These are important questions to answer in future work to maximize the cost-effectiveness of large-scale improvement interventions in primary care.

Finally, it is important to acknowledge the limitations of the approach we have taken as our work to date has adopted a pre–post-test design, without matched controls. As such, we cannot claim causality for the improvements achieved. However, we have developed and refined a complex intervention to support the identification and management of adult patients with CKD in primary care. Future research can target rigorous evaluation of this promising improvement intervention, which encompasses both technological (audit software) and social (behaviour change) components.

## Authors’ contributions

G.H. and K.O. produced the original drafts of the manuscripts; J.Hu. worked as an improvement facilitator, J.He. as the clinical lead and G.H. as the academic lead on the project; K.R. conducted the qualitative evaluation study. All authors contributed to drafts of the manuscript. All authors read and approved the final manuscript.

## Funding

This project is funded by the National Institute for Health Research Collaboration for Leadership in Applied Health Research and Care (NIHR CLAHRC) for Greater Manchester. The views expressed in this article are those of the authors and not necessarily those of the NHS, the NIHR or the Department of Health. Funding to pay the Open Access publication charges for this article was provided by The National Institute for Health Research (NIHR) Collaboration for Leadership in Applied Health Research and Care (CLAHRC) Greater Manchester.
